# Authors’ Response to Peer Reviews of “The Psychological Impact of Hypertension During COVID-19 Restrictions: Retrospective Case-Control Study”

**DOI:** 10.2196/28718

**Published:** 2021-03-30

**Authors:** Carissa Bonner, Erin Cvejic, Julie Ayre, Jennifer Isautier, Christopher Semsarian, Brooke Nickel, Carys Batcup, Kristen Pickles, Rachael Dodd, Samuel Cornell, Tessa Copp, Kirsten J McCaffery

**Affiliations:** 1 Sydney Health Literacy Lab, School of Public Health Faculty of Medicine and Health The University of Sydney Sydney Australia; 2 Agnes Ginges Centre for Molecular Cardiology Centenary Institute The University of Sydney Sydney Australia; 3 Faculty of Medicine and Health The University of Sydney Sydney Australia; 4 Department of Cardiology Royal Prince Alfred Hospital Sydney Australia

**Keywords:** public health, global health, COVID-19, hypertension, risk, strategy, mental health, behavior, response, anxiety, vaccine, retrospective, perception, prevention, intention

*Authors’ response to peer reviews for “The Psychological Impact of Hypertension During COVID-19 Restrictions: Retrospective Case-Control Study.”*

## Response to Reviews

The authors of the manuscript [1] are grateful to the editor and reviewers [2,3] for their invaluable input and feedback.

### Reviewer G

#### Round 1

##### Specific Comments: Major

Thank you for your review [2]. We wrote this as a brief correspondence piece for rapid publication as a preprint because COVID-19 research and public health communications were rapidly evolving at this time. However, we have now rewritten the paper in standard paper format to address your concerns— this includes a much more detailed introduction and rationale, more explanations in the *Methods* section including assumptions, and an expanded discussion. Please see our detailed responses below.

##### Specific Comments: Minor

We have addressed the issues highlighted in the rewritten manuscript. Please see our detailed responses below.

##### Detailed Responses

###### Title/Abstract and References

1. We have changed the title to better reflect our methodology.

2. We have rewritten our work as a full paper rather than a short correspondence, including suggested references and other research that has emerged since the publication of our rapid preprint. The media references are important as they provide context for the study during a rapidly changing COVID-19 response.

###### Introduction

1 and 2. We have included the references/points mentioned by the reviewer and a summary of other research that has emerged since the publication of our rapid preprint. Please note the *New England Journal of Medicine* paper has been retracted so we have not included this.

###### Methods

1. We have included more details on methods to address the reviewer’s points. This includes new sections on the setting, matching, and analysis.

2. We confirm that the original ethics approval covers all subsequent surveys and amendments. We have clarified this in the manuscript.

3. Another preprint from our study [4] that describes the sample at different time point shows it is comparable between April and June. We acknowledge that the respondents who remained in the study were likely more motivated and interested in COVID-19 prevention than those who dropped out. This is mentioned in the *Discussion* section. However, since the study design is not a randomized controlled trial, cases were matched to controls at the same time point, with demographic characteristics controlled for in the analyses, so our key comparison findings should not be affected by those differences.

We have also now performed exploratory analyses of the hypertension subsample by whether they were invited and returned for follow-up, compared to those who were not invited or did not return. We have included a text summary in the paper: “Those who were invited and returned for follow-up were similar for age and gender but had higher levels of education (*P*=.02) and were more likely to have adequate health literacy (*P*=.009).”

4. We have added more details about measures. This followed a US study published in the *Annals of Internal Medicine* [5], with whose authors we are collaborating on an international comparison.

“Participants were asked if they had any of the following conditions: asthma, chronic obstructive pulmonary disease, high blood pressure (hypertension), cancer, heart disease, stroke, diabetes, depression, anxiety.”

5. We have added more details in the new *Methods* section.

6. We have added more details in the new *Methods* section.

7. (a) The estimates referred to as “MMDs” from the linear models are marginal mean differences, not maximum mean discrepancy. This abbreviation is noted in the first row of Table 2. We have now added the abbreviations to the footnote of the table to avoid confusion and clarified the first use of MMD when reporting results in text.

(b) In regard to the use of a modified Poisson approach and reporting of relative risks: with increasing event rates, the difference between an odds ratio (as estimated by logistic regression) and the risk ratio (as estimated from a log-binomial or modified Poisson model) also increases, with odds ratios often incorrectly interpreted as if they are risks. As the reviewer points out, the study design is cross-sectional, so a risk/prevalence ratio is typically considered more appropriate and conceptually easier to interpret than an odds ratio. Although log-binomial regression can also be used to estimate the risk ratio, it is often criticized for producing confidence intervals that are narrower than they should be (ie, due to smaller than expected standard errors) and may also fail to converge. For this reason, we have employed a modified Poisson approach [6], which generates coefficients that, when exponentiated, represent the risk ratio, with corresponding confidence intervals of an appropriate width. As for reporting relative risk, numerically, the risk ratio/relative risk and prevalence ratio are identical, differing only in their interpretation based on the study design. In line with the reviewer’s comment, we have changed the language used and describe the effect as an adjusted prevalence ratio rather than adjusted relative risk to better reflect the study design.

8. We have added a statement on this.

###### Results

1. We have added the footnote. Pairwise comparisons showed no statistically significant differences in age, gender, education, or health literacy between the hypertension and control groups (see the section on matching). We have explained social distancing and patient activation, and clarified the prescription question in the *Methods* section.

2. We have added this.

3. Please see our response above regarding MMD (marginal mean differences from linear regression models). As for the social distancing score, this is a typographical error and has been corrected.

4. We have clarified that these are two separate questions.

5. We have moved this to the *Discussion* section with additional explanations.

6. We have clarified this.

###### Discussion and Conclusions

1-6. We have rewritten the article as a full paper rather than a short correspondence, including a more expansive discussion to address the points mentioned. We have highlighted key findings upfront, discussed different perspectives including access to care, clarified that we only measured vaccination intentions throughout, discussed the implications of the limitations, included points about misinformation on social media, and have more carefully explained our conclusions.

### Reviewer AM

#### Round 1

Thank you for your review [3]. We have addressed the comments as follows.

##### Title

We have revised the title to better reflect the study methods.

##### Abstract

We have revised this as suggested.

##### Methods

1. We have added this.

2. We have expanded the *Methods* section to clarify this.

##### Results

1. Our controls were defined as not having comorbidities thought to be relevant to COVID-19 outcomes at the time of the study. Other medications (eg, contraception or unrelated conditions) could have been taken, but we did not ask for these details in this survey.

2. Per our previous responses, we have now expanded our *Methods* and *Statistical Analysis* sections, clarifying that the MMD was calculated for continuous outcomes using linear regression models.

Although the risk perception measure has a restricted range of 0-10, the normality assumption of a general linear model relates to the distribution of the residuals (which should also be homogenous across the fitted values). These assumptions were explored graphically (via a histogram of residuals with superimposed normal density and a plot of the residuals against the fitted values with a superimposed smoothed lowess line), and was deemed to be sufficiently satisfied.

Notably, an alternative analysis approach for ordinal Likert-scale data would be to apply an ordinal logistic regression model. We have explored this option given the reviewer’s comment; this elicited comparable outcomes. However, there was substantially more difficulty associated with the interpretation given the outcome of such a model is the adjusted odds ratio of responding one unit higher on the response scale for cases relative to controls. As such, we feel the application of linear regression remains a more suitable option for this outcome variable.

3. We have added this.

##### Discussion/Limitations

We have included a more expansive introduction and discussion to address the reviewer’s points. Our COVID-19 risk perception and vaccination intention scores were very high across groups, indicating a possible ceiling effect, but this is consistent with other Australian surveys, which is explained in our discussion. We have explained why we isolated the effect of hypertension from other comorbidities in the expanded paper with more details in the *Introduction* and *Methods* sections.

#### Round 2

We have added the ethics review/approval number in this version of the manuscript as requested.
